# Elevated Urinary Rab10 Phosphorylation in Idiopathic Parkinson Disease

**DOI:** 10.1002/mds.29043

**Published:** 2022-05-06

**Authors:** Shijie Wang, Shakthi Unnithan, Nicole Bryant, Allison Chang, Liana S. Rosenthal, Alexander Pantelyat, Ted M. Dawson, Hussein R. Al‐Khalidi, Andrew B. West

**Affiliations:** ^1^ Duke Center for Neurodegeneration and Neurotherapeutics Duke University Durham North Carolina USA; ^2^ Department of Biostatistics and Bioinformatics Duke University Durham North Carolina USA; ^3^ Department of Neurology The Johns Hopkins University Baltimore Maryland USA; ^4^ Neurodegeneration and Stem Cell Programs, Institute for Cell Engineering Johns Hopkins University School of Medicine Baltimore Maryland USA; ^5^ Solomon H. Snyder Department of Neuroscience Johns Hopkins University School of Medicine Baltimore Maryland USA; ^6^ Department of Pharmacology and Molecular Sciences Johns Hopkins University School of Medicine Baltimore Maryland USA

**Keywords:** biomarker, Park8, longitudinal, neurodegeneration

## Abstract

**Background:**

Pathogenic *leucine‐rich repeat kinase 2 LRRK2* mutations may increase LRRK2 kinase activity and Rab substrate phosphorylation. Genetic association studies link variation in *LRRK2* to idiopathic Parkinson disease (iPD) risk.

**Objectives:**

Through measurements of the LRRK2 kinase substrate pT73‐Rab10 in urinary extracellular vesicles, this study seeks to understand how LRRK2 kinase activity might change with iPD progression.

**Methods:**

Using an immunoblotting approach validated in LRRK2 transgenic mice, the ratio of pT73‐Rab10 to total Rab10 protein was measured in extracellular vesicles from a cross‐section of G2019S *LRRK2* mutation carriers (N = 45 participants) as well as 485 urine samples from a novel longitudinal cohort of iPD and controls (N = 85 participants). Generalized estimating equations were used to conduct analyses with commonly used clinical scales.

**Results:**

Although the G2019S *LRRK2* mutation did not increase pT73‐Rab10 levels, the ratio of pT73‐Rab10 to total Rab10 nominally increased over baseline in iPD urine vesicle samples with time, but did not increase in age‐matched controls (1.34‐fold vs. 1.05‐fold, 95% confidence interval [CI], 0.004–0.56; *P* = 0.046; Welch's *t* test). Effect estimates adjusting for sex, age, disease duration, diagnosis, and baseline clinical scores identified increasing total Movement Disorder Society‐Sponsored Revision of the Unified (MDS‐UPDRS) scores (β = 0.77; CI, 0.52–1.01; *P* = 0.0001) with each fold increase of pT73‐Rab10 to total Rab10. Lower Montreal Cognitive Assessment (MoCA) score in iPD is also associated with increased pT73‐Rab10.

**Conclusions:**

These results provide initial insights into peripheral LRRK2‐dependent Rab phosphorylation, measured in biobanked urine, where higher levels of pT73‐Rab10 are associated with worse disease progression. © 2022 The Authors. *Movement Disorders* published by Wiley Periodicals LLC on behalf of International Parkinson Movement Disorder Society.

Recent genome sequencing efforts have found that missense mutations in the *leucine‐rich repeat kinase 2* (*LRRK2*) gene, in particular G2019S, are one of the most prevalent genetic causes of Parkinson disease (PD).^1^, ^2^ Pathogenic *LRRK2* mutations induce an apparent gain‐of‐function in the encoded LRRK2 protein with respect to cis autophosphorylation,^3^, ^4^ as well as increased trans phosphorylation of other Rab proteins including Rab8a, Rab10, Rab12, Rab29, and Rab35.^5^, ^6^ The Rab10 LRRK2 kinase substrate has been studied in model systems owing in part to the recent availability of highly‐specific monoclonal pT73‐Rab10 and total Rab10 antibodies that do not appear to significantly cross‐react with other similar Rab proteins (ie, Rab8a).^7^ LRRK2 phosphorylation of Rab10 may bolster pro‐inflammatory immunological responses in immune cells^7^ and impair ciliogenesis in neurons.^8^ The existence of other as yet unknown functions in cells that express both LRRK2 and Rab10 seem highly likely.

Recent studies have implicated elevated LRRK2 activity in idiopathic PD (iPD cases, without LRRK2 mutations) in post‐mortem tissue studies.^9^ Acknowledging the widespread expression of both LRRK2 and Rab10 across the body, measurements of the LRRK2 kinase substrate Rab10 in biofluids from well clinically‐characterized subjects may provide further insight into whether LRRK2 kinase activity may be deregulated in some iPD cases. Studies with human neutrophils and monocytes isolated from blood and treated with LRRK2 kinase inhibitors have demonstrated that pT73‐Rab10 levels are dependent on LRRK2 kinase activity.^10^, ^11^ However, in mouse embryonic fibroblasts, the G2019S *LRRK2* mutation had only a modest effect on pT73‐Rab10 levels (~1.4‐fold increase vs. WT‐LRRK2) compared to a larger effect noted for the R1441C *LRRK2* mutation (~2.5‐fold increase vs. WT‐LRRK2).^12^, ^13^ In a recent study, we successfully measured pT73‐Rab10 in macaque urine extracellular vesicle fractions using a Western blot approach.^14^ The ratio of pT73‐Rab10 to total Rab10 in the macaque urine diminished after treatment with highly selective LRRK2 kinase inhibitors. Total Rab10 levels did not appear sensitive to LRRK2 expression or activity. In human samples from iPD and controls, our past proteomic work corroborated the presence of high levels of total Rab10 protein in human urine extracellular vesicle fractions that were relatively stable over time, but not different in total Rab10 concentration between iPD cases and controls.^15^

Here, we measured pT73‐Rab10 levels in urine from a longitudinal cohort of iPD and controls for potential interactions with clinical phenotypes. In a smaller cross‐sectional series, we also measured the effects of the G2019S *LRRK2* mutation. Our findings suggest that increased pT73‐Rab10 levels in urine, which gradually increased over time in some iPD cases, are associated with worse disease. However, the G2019S *LRRK2* mutation does not appear to further upregulate pT73‐Rab10, at least in urinary extracellular vesicles. These results provide some of the first insights into how LRRK2 activity in the periphery, as measured in urine, might change with disease progression.

## Methods

### Description of the Michael J. Fox Foundation for Parkinson's Disease Research LRRK2 Cohort Sample Distribution

Urine samples approved for distribution were provided with coded identifiers in ~10 mL aliquots and distributed via the National Institute of Neurological Disorders and Stroke (NINDS) Biorepository at Indiana University. The Michael J. Fox Foundation for Parkinson's Disease Research LRRK2 Cohort (MJFF‐LCC) is coordinated and funded by The Michael J. Fox Foundation for Parkinson's Research. For up‐to‐date information on the study, please visit https://www.michaeljfox.org/biospecimens. Participants from this distribution did not overlap with subjects previously analyzed by our group according to available unique subject identifiers.^4^, ^17^, ^18^ Samples from males‐only were approved for distribution for this study, because of limited availability of samples from females. In total, measurements were made from 45 coded urine samples from this study group (Supplementary Table S1), and samples were assigned to groups after final data curation was provided to the MJFF‐LCC.

### Description of the Johns Hopkins Parkinson Disease Biomarker Program Study Population

Biobanked urine samples were collected from iPD patients (screened negative for known pathogenic mutations through the PDBP consortium) and neurologically healthy controls, recruited into a biomarker surveillance program established at Johns Hopkins University as part of the larger NINDS Parkinson's disease Biomarker Program (JH‐PDBP). A total of 127 study participants, including 90 cases and 37 controls, were enrolled in the surveillance program between 2013 and 2018 (Supplementary Table S2). Urine donation was optional in the study, and subjects who donated at least three viable urine specimens (at least 40 mL) from at least three separate study visits that spanned at least 1.5 years were included in the study group. In total, 66 PD cases and 19 controls met criteria (n = 85) (Table 1). Therefore, 24 PD cases were excluded (27%) and 18 controls were excluded (49%). The Movement Disorder Society‐Sponsored Revision of the Unified (MDS‐UPDRS) and Montreal Cognitive Assessment (MoCA) were assessed and recorded for all subjects, and levodopa equivalent daily dosage (LEDD)^16^ were calculated and recorded for each study visit. Additional clinical information is available online (https://pdbp.ninds.nih.gov/). In total, from the JH‐PDBP study group, it was possible to measure both LRRK2 protein and the ratio of phosphorylated to total Rab10 in 485 coded urine samples. Sample identifiers were unblinded after final data curation for the measurements of the target proteins, as described below.

**TABLE 1:** JH‐PDBP study participant baseline characteristics after subject exclusion

Characteristic	Case	Control	Total
Age (y)			
N	66	19	85
Mean (SD)	66.4 (7.3)	65.6 (8.9)	66.2 (7.6)
Median (q1,q3)	67.2 (61.7, 71.2)	66.4 (57.3, 70.3)	67.2 (61.1, 70.3)
Min/max	49.2/88.0	46.1/82.5	46.1/88.0
Age at PD diagnosis (y)			
N	66	NA	66
Mean (SD)	59.6 (8.4)	NA	59.6 (8.4)
Median (q1,q3)	60.0 (54.0, 66.0)	NA	60.0 (54.0, 66.0)
Min/max	40.0/82.0	NA	40.0/82.0
PD duration (y)			
N	66	NA	66
Mean (SD)	6.9 (4.8)	NA	6.9 (4.8)
Median (q1,q3)	5.4 (3.3, 8.9)	NA	5.4 (3.3, 8.9)
Min/max	1.0/25.5	NA	1.0/25.5
Female	19/66 (28.8%)	12/19 (63.2%)	31/85 (36.5%)
Male	47/66 (71.2%)	7/19 (36.8%)	54/85 (63.5%)
Education			
High School, diploma	6 (9.1%)	0 (0.0%)	6 (7.1%)
Some college, no degree	6 (9.1%)	4 (21.1%)	10 (11.8%)
College degree	22 (33.3%)	6 (31.6%)	28 (32.9%)
Master's degree	23 (34.8%)	4 (21.1%)	27 (31.8%)
Doctoral degree	4 (6.1%)	2 (10.5%)	6 (7.1%)
Professional degree	5 (7.6%)	3 (15.8%)	8 (9.4%)
LEDD			
N	66	NA	66
Mean (SD)	768.3 (489.6)	NA	768.3 (489.6)
Median (q1,q3)	600.0 (475.0, 1000.0)	NA	600.0 (475.0, 1000.0)
Min/max	50.0/2318.0	NA	50.0/2318.0
MDS‐UPDRS Part I score:			
N	63	19	82
Mean (SD)	11.3 (5.4)	5.3 (4.8)	9.9 (5.8)
Median (q1,q3)	10.0 (8.0, 13.0)	3.0 (2.0, 8.0)	9.5 (7.0, 12.0)
Min/max	2.0/29.0	1.0/18.0	1.0/29.0
MDS‐UPDRS Part II score			
N	63	19	82
Mean (SD)	11.8 (6.9)	0.9 (1.9)	9.3 (7.6)
Median (q1,q3)	10.0 (7.0, 16.0)	0.0 (0.0, 1.0)	9.0 (3.0, 13.0)
Min/Max	2.0/35.0	0.0/7.0	0.0/35.0
MDS‐UPDRS total score			
N	63	19	82
Mean (SD)	58.3 (22.8)	7.7 (7.6)	46.6 (29.5)
Median (q1,q3)	55.0 (45.0, 64.0)	4.0 (3.0, 10.0)	50.0 (29.0, 61.0)
Min/max	22.0/137.0	1.0/31.0	1.0/137.0
MoCA Total			
N	66	19	85
Mean (SD)	26.3 (3.2)	28.2 (1.1)	26.7 (2.9)
Median (q1,q3)	27.0 (25.0, 29.0)	28.0 (27.0, 29.0)	27.0 (25.0, 29.0)
Min/max	13.0/30.0	26.0/30.0	13.0/30.0
S&E score			
N	65	19	84
Mean (SD)	1.7 (1.3)	0.1 (0.2)	1.3 (1.3)
Median (q1,q3)	1.0 (1.0, 2.0)	0.0 (0.0, 0.0)	1.0 (1.0, 2.0)
Min/max	0.0/7.0	0.0/1.0	0.0/7.0
Total LRRK2			
N	65	19	84
Mean (SD)	17.1 (73.5)	36.0 (76.3)	21.4 (74.1)
Median (q1,q3)	4.8 (1.3, 10.8)	2.5 (1.3, 7.8)	3.1 (1.3, 10.1)
Min/max	0.1/593.3	0.4/297.3	0.1/593.3
pT73‐Rab10/Total Rab10			
N	66	19	85
Mean (SD)	1.1 (0.9)	1.3 (0.9)	1.1 (0.9)
Median (q1,q3)	0.9 (0.6, 1.3)	1.0 (0.6, 2.1)	0.9 (0.6, 1.3)
Min/max	0.3/6.4	0.3/3.3	0.3/6.4

Abbreviations: LEDD, L‐dopa equivalent; MDS‐UPDRS, Movement Disorder Society‐Sponsored Revision of the Unified. Parkinson's Disease Rating Scale; MoCA, The Montreal Cognitive Assessment; S&E, Schwab and England Scale.

### Determination of the Ratio of pT73‐Rab10 to Total Rab10 in Urine Samples

Biobanked urine samples, collected and stored frozen in standard polypropylene tubes, were processed as previously described.^4^, ^17^, ^18^ After quick‐thaw, urine fractions enriched in extracellular vesicles were isolated through differential ultracentrifugation including a first round clear at 10 000 × *g* spin at 30 minutes at 4°C, pellet discarded, and a second round 100 000 × *g* spin for 1 hour at 4°C. Resultant “P100” pellets were washed in 1 mL phosphate‐buffered saline (PBS) and directly lysed in Laemmli sample buffer freshly prepared (2% sodium dodecyl sulfate [SDS], 10% glycerol, 40 mM NaF, 60 mM Tris‐CL, pH 6.8, and 5% dithiothreitol) to prevent degradation of phosphoproteins and for preservation of the samples at −80°C. A common pooled sample was generated by combining 10% w/v of all samples from either the JH‐PDBP cohort or the MJFF‐LCC cohort. The creation of the JH‐PDBP sample pool and analysis of JH‐PDBP samples occurred more than a year apart from the creation of the MJFF‐LCC sample pool and analysis of MJFF‐LCC samples, and there were no attempts made to compare protein concentrations in the two different pools. Approximately 1 μg of protein from the P100 fractions from each sample were loaded to 4%–20% gradient mini‐PROTEAN TGX stain‐free gels (BioRad, Hercules, CA) and transferred to Immobilon‐PVDF membranes (Millipore, St. Loius, MO, followed by immunoblotting with N241/34 anti‐LRRK2 (Antibodies Inc., Davis, CA) in the upper half of the membrane, or phospho‐T73‐Rab10 (MJF‐R21, Abcam, Fremont, CA) or total Rab10 antibody (MJF‐R23, Abcam, in the lower half of the membrane, with goat anti‐rabbit or anti‐mouse HRP (Jackson Immuno, West Grove, PA). Samples were typically analyzed over three runs to derive mean values compared to the sample pool. All signals (Crescendo reagent, Millipore) were captured digitally (Chemidoc MP, BioRad) and quantifications were automated (Image Lab 6.0.1, BioRad). Samples were processed in randomized bulks without respect to group assignment or collection dates, with the same common sample pool present with every independent analysis and run.

### Mouse Kidney Lysate Analysis

For validation of the antibodies and the ratio of pT73‐Rab10 to total Rab10 as a function of LRRK2 activity, we used a cohort of mice with genetically altered LRRK2 kinase activity and/or expression including C57BL/6J (JAX stock 000664), LRRK2 R1441C‐KI (JAX Stock 009346), LRRK2 G2019S‐KI (JAX Stock 018785), LRRK2 G2019S‐BAC (JAX stock 012467), and LRRK2 knockout mice that have no LRRK2 kinase activity or expression (JAX Stock 016121). Adult male mice were perfused with cold PBS and both left and right kidneys were dissected into the Laemmli sample buffer described above. As samples fit on a single blot, Hsc70 measurements, instead of a pooled‐sample control, were made with an Hsc70 monoclonal antibody from Cell Signaling (Danvers, MA).

### Statistical Analysis

Subject clinical and demographic characteristics at baseline in the JH‐PDBP pool (first study visit) or MJFF‐LCC were summarized as means (standard deviation [SD]), medians (25th and 75th percentiles), min and max for continuous variables, and as counts (percentages) for categorical variables in Table 1. Statistical hypotheses were tested as two‐sided at 0.05 level of significance. Generalized estimating equations (GEEs) models were used to conduct longitudinal analyses in the JH‐PDBP data for four selected outcomes typically used to measure PD progression in mid to late‐stage disease in clinical trials^19^, ^20^, ^21^: MDS‐UPDRS Part I Score, MDS‐UPDRS Part II Score, MDS‐UPDRS Total Score, and MoCA score. Owing to the sensitivity of MDS‐UPDRS Part III scores to dopamine replacement therapies in mid‐stage iPD and interactions of Part III with LEDD, we opted to not include MDS‐UPDRS Part III separately in the analysis, opting to instead focus on biomarker interactions with MDS‐UPDRS Total Scores. Two models examined each outcome including the main effect and interaction effect model for the ratio of pT73‐Rab10 to total Rab10. Both models were adjusted for gender, age, disease duration, the patient's baseline score, and case–control status. Significance was determined at α = 0.05.

The main effects model is indicated below:

$$\text{YScore}=\beta_{0}+\beta_{1}\left(\mathrm{Age}\right)+\beta_{2}\left(\text{Gender}\right)+\beta_{3}\left(\text{Disease Duration}\right)+\beta_{4}\left(\text{Control}\right)+\beta_{5}\left(\text{Baseline Score}\right)+\left(\mathrm{pT}73/\text{Total}\;\mathrm{Rab}10\right)$$

The interaction effects model is indicated below:

$$\text{YScore}=\beta_{0}+\beta_{1}\left(\mathrm{Age}\right)+\beta_{2}\left(\text{Gender}\right)+\beta_{3}\left(\text{Disease Duration}\right)+\beta_{4}\left(\text{Control}\right)+\beta_{5}\left(\text{Baseline Score}\right)+\beta_{6}\left(\mathrm{pT}73/\text{Total}\;\mathrm{Rab}10\right)+\beta_{7}\left(\mathrm{pT}73/\text{Total}\;\mathrm{Rab}10*\text{Control}\right)+\beta_{8}\left(\mathrm{pT}73/\text{Total}\;\mathrm{Rab}10*\text{Gender}\right)$$

Additionally, the models for the MoCA score were adjusted for ApoE genotype status. For both models, a compound symmetry structure was used as a working covariance matrix. Robust standard errors were used to construct the 95% confidence intervals (CIs) around estimated model coefficients. Correlations were evaluated using Pearson's r, and the mouse kidney lysate dataset and MJFF‐LCC dataset were analyzed by one‐way ANOVA and post‐hoc tests as described.

## Results

### The pT73‐Rab10 to Total Rab10 Ratio is Modulated by LRRK2 Activity

**FIG 1:**
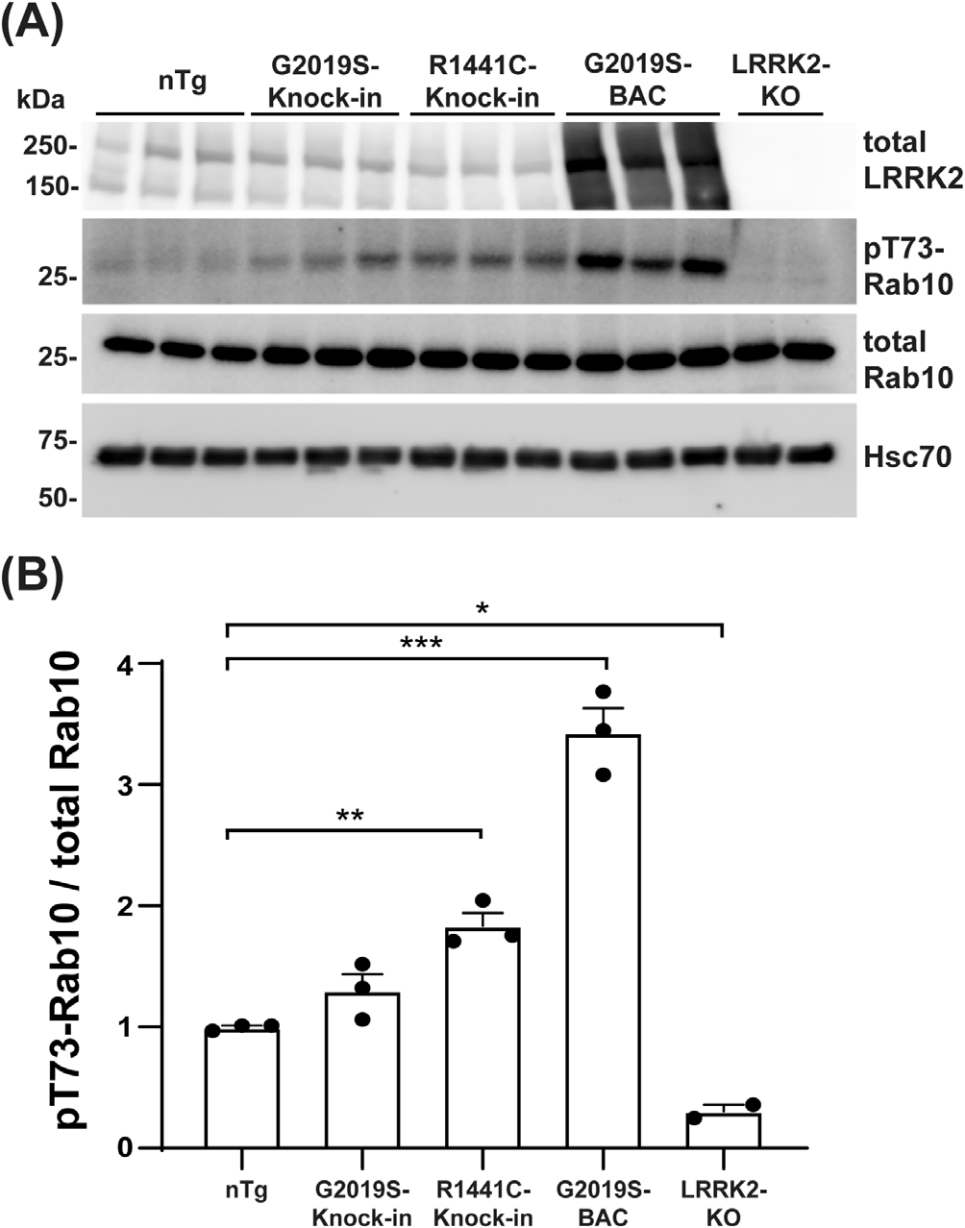
Analysis of the ratio of pT73‐Rab10 to total Rab10 protein in mouse kidney lysates. Kidney lysates were generated from mice with the indicated genotype that include 4‐ to 6‐month‐old male non‐transgenic (nTg) C57BL/6J mice, homozygous G2019S‐knockin *LRRK2* mice, homozygous R1441C‐knockin *LRRK2* mice, transgenic G2019S‐mouse‐BAC *LRRK2* mice, and *LRRK2* homozygous knockout mice. (**A**) Representative immunoblots for the indicated protein target (total LRRK2, pT73‐Rab10, total Rab10, and the housekeeping protein Hsc70). (**B**) Quantification of pT73‐Rab10 (normalized to Hsc70 in the same lane of the membrane) as a ratio with total Rab10 (also normalized to Hsc70 in the same lane), where a ratio of 1.0 represents the mean value of the trio of nTg mice analyzed here. Each data point represents the mean analysis of a mouse lysate, combined from left and right kidney analysis. Significance is assessed by one‐way ANOVA, and results from Tukey's post‐hoc test are indicated with *P < 0.05, **P < 0.01, ***P < 0.001.

Previously, using an immunoblotting approach, we identified a lower ratio of pT73‐Rab10 to total Rab10 protein in macaque urine extracellular vesicle lysates after the animals were treated for 1 week with the selective LRRK2 kinase inhibitor MLi2 or the structurally distinct LRRK2 inhibitor PFE‐360.^22^ To help validate the approach as sensitive to increases in LRRK2 kinase activity, we procured total protein lysates from the kidneys of non‐transgenic mice, mice homozygous for a G2019S knockin mutation, mice homozygous for an R1441C‐Lrrk2 knockin mutation; G2019S‐BAC *LRRK2* mice that over‐express G2019S‐LRRK2 ~10‐fold over endogenous levels,^23^, ^24^ and *LRRK2* knockout mice.^25^ Kidney lysates were used in lieu of urine as we could not collect enough mouse urine to meet the lower limits of sensitivity of the assay (typically 10 mL needed). In the kidney lysates, total Rab10 expression was similar across all mouse strains, consistent with LRRK2 activity not modifying total Rab10 expression (ie, through phosphorylation) as observed in other tissues and cell types.^6^, ^7^ The G2019S LRRK2 knockin lysates were similar to those from non‐transgenic (nTg) lysates with respect to total LRRK2 expression and pT73‐Rab10 levels, consistent with past reports in other tissues.^5^, ^6^ A ~3.5‐fold increase in the pT73‐Rab10 to total Rab10 ratio with G2019S‐BAC LRRK2 overexpression was observed, presumably because of the higher total LRRK2 protein expression (Fig. 1B). The R1441C homozygous knockin mutation was associated with a ~1.8‐fold increase in the ratio of pT73‐Rab10 to total Rab10 protein, whereas the LRRK2 knockout had nearly undetectable levels of pT73‐Rab10 compared to lysates from nTgs. These results help establish the measured ratio in the periphery as dynamic with respect to LRRK2 activity, although the effect specifically of the G2019S *LRRK2* mutation is not clear.

### The pT73‐Rab10 to Total Rab10 Ratio in Urine Does Not Discriminate G2019S *LRRK2* Mutation Carriers

**FIG 2:**
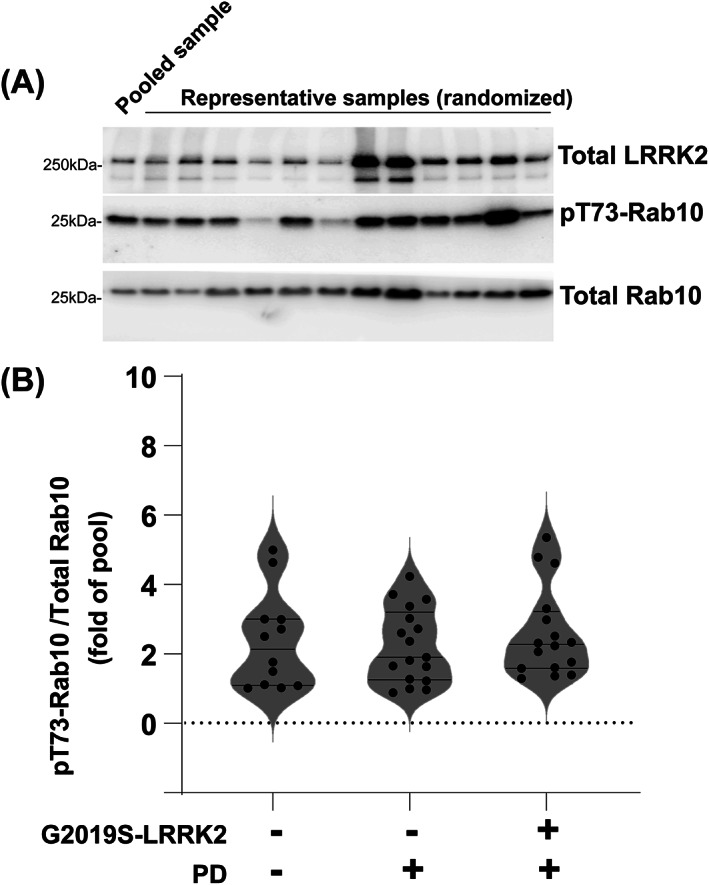
G2019S‐*LRRK2* carriers do not demonstrate elevated pT73‐Rab10 levels in urine biobanked from a cross‐sectional cohort. Urine samples from male participants collected as part of the MJFF‐LCC sample series were processed into P100 exosome‐enriched pellets, and the ratio of pT73‐Rab10 to total Rab10 was compared to the sample pool. (**A**) Representative immunoblots of randomized samples and the “pooled sample” reference. The pool is composed of ~10% w/v of all samples in the study and was created independently of the JH‐PDBP sample pool. (**B**) Quantification of pT73‐Rab10 to total Rab10 ratio from the cohort, with the ratio of pT73‐Rab10 to total Rab10 in the pool set to 1.0 (one‐way ANOVA *P* > 0.5). Violin plots and bars highlight mean group values and ±SEM.

To help determine whether G2019S *LRRK2* mutation carriers harbor increased levels of pT73‐Rab10 in urine, we obtained a 45 sample cross‐section of samples from the MJFF‐LCC (Supplementary Table S1). Samples assigned to three groups, healthy controls, idiopathic PD, and G2019S‐*LRRK2* carriers with PD, had a similar mean age and were all male participants. Disease duration of 9.5 years on average in the mutation carrier group was longer than the PD group in the JH‐PDBP cohort at baseline (6.9 years), with a corresponding worse MoCA score distribution in the *LRRK2* mutation group than the JH‐PDBP group (Supplementary Table S2). In this cohort, the ratio of pT73‐Rab10 to total Rab10 compared to the pool (from the MJFF‐LCC samples) (Fig. 2A) was similar between carriers and non‐carriers, in cases and controls (one‐way ANOVA *P* = 0.36) (Fig. 2B).

### Increased Urinary pT73‐Rab10 to Total Rab10 Ratios in iPD Progression

**FIG 3:**
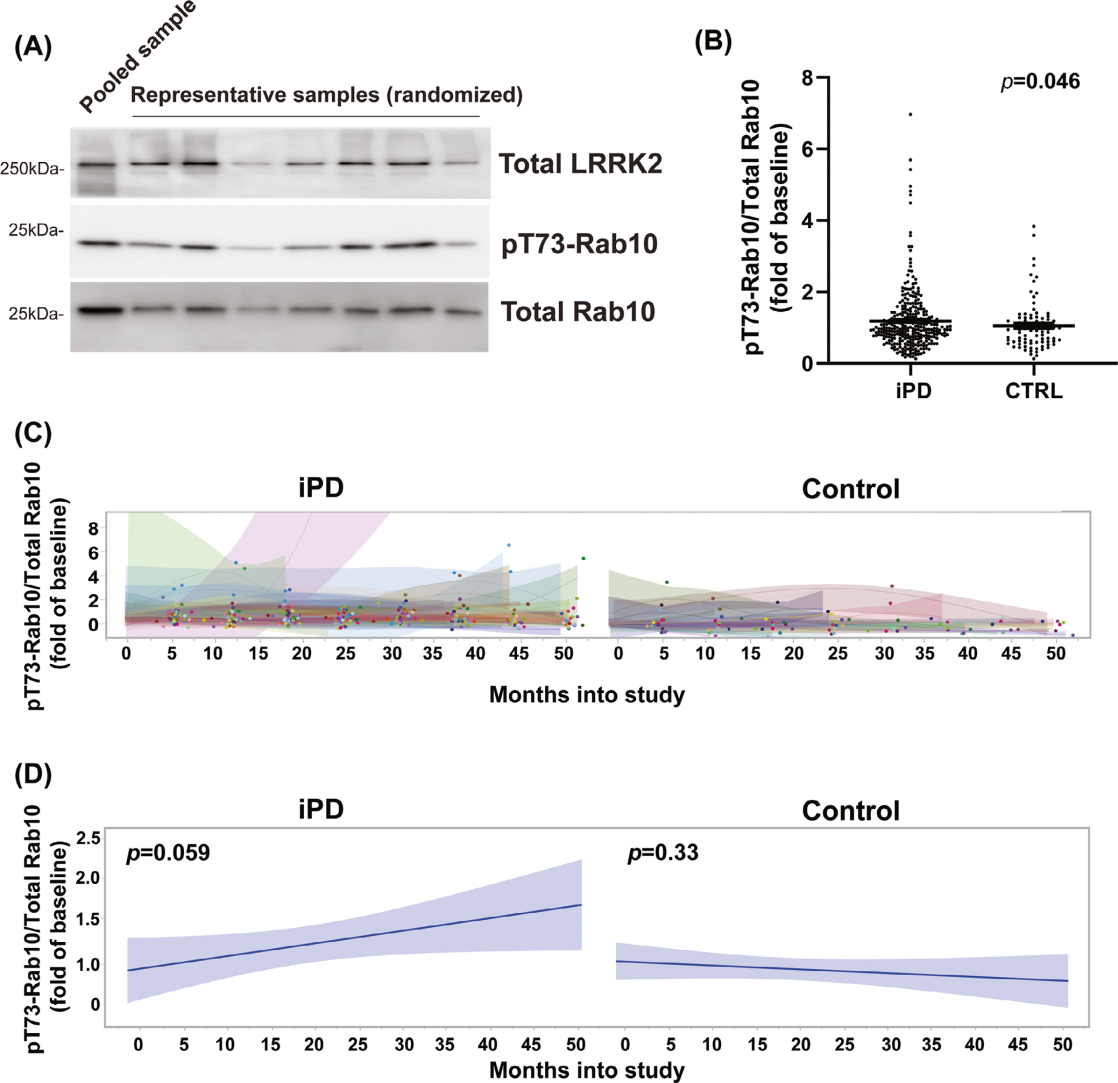
Longitudinal trajectory of the change in the ratio of pT73‐Rab10 to total Rab10 over time in idiopathic PD (iPD) and healthy controls. pT73‐Rab10, total Rab10, and total LRRK2 proteins were measured by Western blot from the ultracentrifugation pellet (P100) from biobanked urine. (**A**) Representative immunoblots show randomized samples from the JH‐PDBP series compared to the JH‐PDBP pool sample analyzed on all runs. The pool sample is composed of ~10% w/v of all samples from the JH‐PDBP cohort. Measures from all blots from all samples are calculated as a percentage of the concentration of the pooled sample, with the pT73‐Rab10 to total Rab10 ratio subsequently derived. (**B**) Scatter plot of pT73‐Rab10 to total Rab10 ratio changes (unadjusted for demographics or clinical scores) with the baseline ratio (first measurement, or “month 0”) set as 1.0 for each subject, and subsequent ratios calculated as a fold change of the baseline ratio for that subject. The *P* value in B was calculated using Welch's *t* test, with unequal variance between cases and controls (ie, higher variance in iPD, *F*[312,85] = 9.5, *P* < 0.0001). Each data point represents a calculated pT73‐Rab10 to total Rab10 ratio change from baseline, with baseline (set at 1.0 for all subjects) not included in the statistical tests. Lines indicate geometric means with 95% CI error bars. (**C**) Longitudinal trajectory of each subject, and (**D**) the mean change in the ratio of pT73‐Rab10 to total Rab10. Two dots that are high (31.4 and 18.0 in the iPD group) are not shown in the plots. Shaded areas in the plots represent 95% CI; *P* values in (**D**) are with respect to linear regression. [Color figure can be viewed at wileyonlinelibrary.com]

To assess the pT73‐Rab10 to total Rab10 ratio in a broader cohort of iPD, urine samples were processed from a longitudinally followed JH‐PDBP study population (Table 1). The average age of 66.4 years in the iPD group was similar to the control group at 65.6 years of age, although the proportion of males in the PD group (71%) was much higher than the proportion of males in the control group (37%), similar to the imbalance in the whole JH‐PDBP cohort (Supplementary Table S2). LEDD at baseline (768 ± 490) corresponded to a mean disease duration of 6.9 ± 4.8 years at the time of baseline (ie, first) sample donation, with an average age at diagnosis of 59.6 ± 8.4 years at baseline. Biobanked frozen urine samples were processed in parallel at the end of the study, a sample pool was created and levels of pT73‐Rab10, total Rab10, and total LRRK2 were compared in abundance to the cohort pool ratio (Fig. 3A). Through study, raw measures of the pool standard that reflect technical variation were 17.5% SD and 12.8% SD for pT73‐Rab10 and total Rab10, respectively (Supplementary Fig. S1). Unadjusted baseline levels of the pT73‐Rab10 to total Rab10 ratio were similar between iPD and controls (two‐sided *t* test, *P* = 0.52) (Table 1). Total LRRK2 levels measured in all samples did not correlate with the ratio of pT73‐Rab10 levels to total Rab10 levels (r = −0.05; *P* = 0.3).

In the analysis of samples across the study period, we noticed an accumulation of samples above a 2‐fold increase (relative to the pool sample) in the ratio of pT73‐Rab10 to total Rab10 particularly at later time points in the iPD group. To better understand how the ratio of pT73‐Rab10 to total Rab10 might change over time, the baseline ratio value was set at 1.0 for each subject with all other time points calculated as a fold change from the baseline. A nominal increase was found in iPD compared to controls (*P* = 0.046; Welch's *t* test) (Fig. 3B). There was markedly more variability in the iPD group compared with controls (*F* test; *P* < 0.0001) (Supplementary Fig. S1), with a possible upward linear trend of rising pT73‐Rab10 to total Rab10 ratios with time in iPD (*P* = 0.059) (Fig. 3C,D). Aside from disease progression, the observed increases could plausibly be explained by increased medication usage with time or increases in males over time (enriched in the iPD group), but not females (under‐represented in the iPD group).

**FIG 4:**
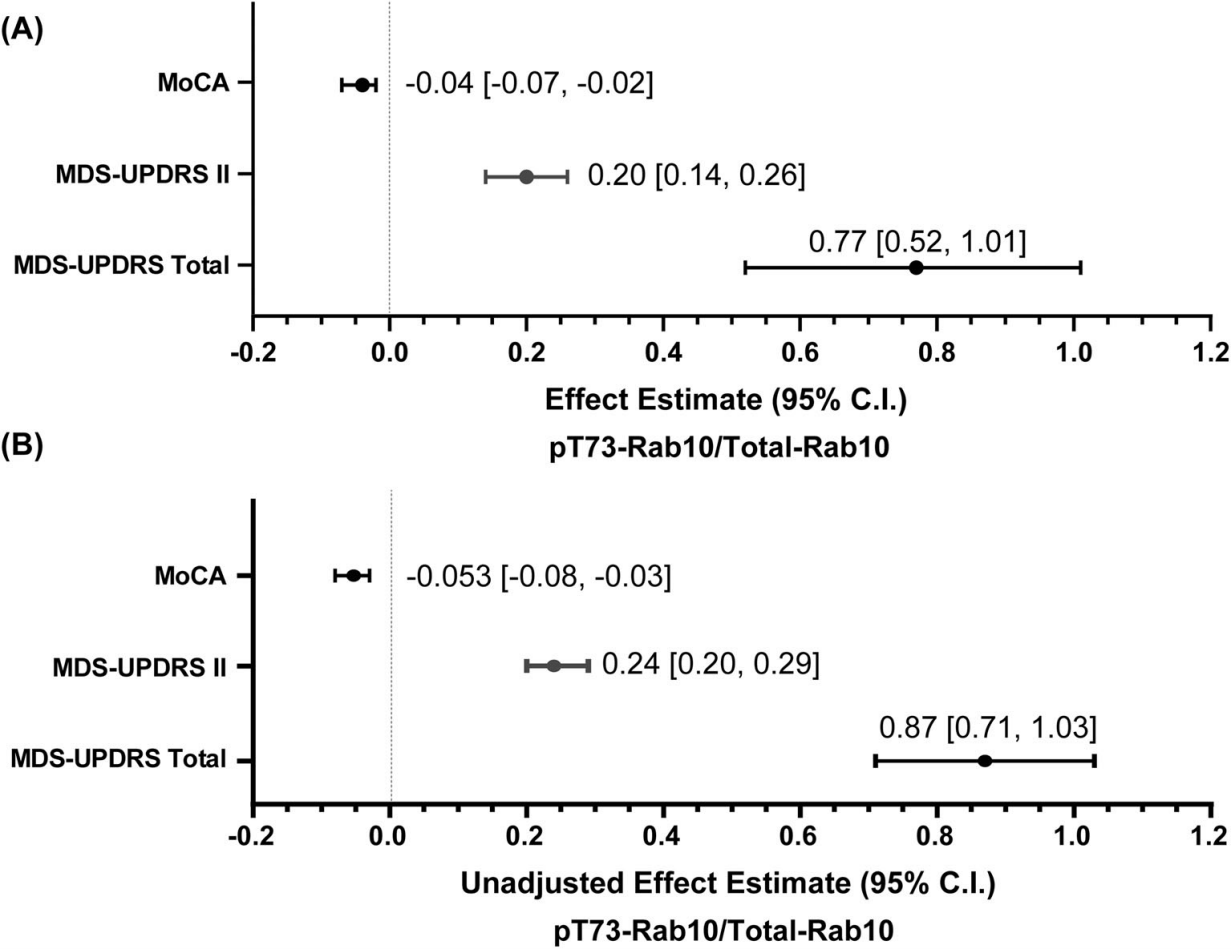
Main effect estimates for the pT73‐Rab10 to total Rab10 ratio and clinical scores. Estimates of a 1‐fold increase (relative to the reference cohort pooled sample) in the pT73‐Rab10 to total Rab10 ratio on MoCA scores, MDS‐UPDRS Part II scores, and total MDS‐UPDRS scores. (**A**) Effect estimates and 95% CI derived from the generalized estimating equation (GEE) that were adjusted for sex, age, disease duration, diagnosis, and baseline clinical scores. The model for the MoCA score was additionally adjusted for ApoE genotypes. (**B**) Corresponding unadjusted effect estimates from the same GEE model.

To better explore these variables, we used GEEs to produce coefficient estimates at 95% CIs. Adjustments included sex, age, disease duration, diagnosis, and baseline clinical score. Disease progression negatively affected the MoCA score in this study group, with an average decrease of 0.13 points per year (β = −0.13; CI, −0.21 to −0.04, *P* = 0.0036). We observed a negative association between the pT73‐Rab10 to total Rab10 protein ratio and MoCA scores (β = −0.04; CI, −0.07 to −0.02; *P* = 0.0008). In the whole cohort, a one‐fold increase (relative to the pool) of the ratio of pT73‐Rab10 to total Rab10 was associated with a small, but statistically significant decrease of 0.04 points (Fig. 4A). This association was not significantly different between the iPD and control group (*P* = 0.84), although the control group had fewer participants. In cases and controls in this study group, ApoE ε4 status did not significantly affect the MoCA scores (ε4 vs. ε2, *P* = 0.75; ε4 vs. ε3, *P* = 0.12), though ApoE genotypes were included in the GEE model. The effect estimate was similar without adjustment (Fig. 4B).

The total MDS‐UPDRS scores increased with disease duration as expected (β = 0.93; CI, 0.24–1.62; *P* = 0.01), similar to changes observed in other mid‐disease iPD cohorts followed for more than 3 years.^26^ Total MDS‐UPDRS scores increased by 0.77 points (CI, 0.52–1.01; *P* < 0.0001) with every fold increase of the pT73‐Rab10 to total Rab10 ratio relative to the study pool (Fig. 4A). Effect estimates were similar without adjustments (Fig. 4B). Within the MDS‐UPDRS, significant interactions were noted in the Part I and Part II MDS‐UPDRS subscales with the ratio of pT73‐Rab10 to total Rab10 ratio. Part I did not significantly increase with disease duration (β = 0.12; CI, −0.02–0.27; *P* = 0.1), whereas Part II increased with disease duration (β = 0.65; CI, 0.43–0.86; *P* < 0.0001). Part I scores are increased by 0.34 points (CI, 0.32–0.37; *P* < 0.0001) with every fold increase of the pT73‐Rab10 to total Rab10 ratio, whereas Part II scores are increased by 0.2 points (CI, 0.14–0.26; *P* < 0.0001). Corresponding unadjusted effect estimates from the same GEE model was similar to the adjusted effect estimates (Fig. 4B).

## Discussion

Genome‐wide association studies in late‐onset iPD highlight the *LRRK2* gene in disease risk.^27^, ^28^ However, the role of LRRK2 in disease progression is less clear. Few studies have biochemically assessed LRRK2 activity markers in biofluids from iPD cases, and no previous study to our knowledge has attempted to measure LRRK2 kinase activity markers longitudinally. Previously, we found that the pT73‐Rab10 to total Rab10 ratio in macaque urine markedly reduced with LRRK2 kinase inhibition. Here, we find that phospho‐Rab10 elevates in mouse kidney lysates with the expression of pathogenic LRRK2 protein and reduces with LRRK2 knockout. Although the G2019S *LRRK2* mutation does not appear to specifically drive the phospho‐Rab10 ratio, we found that individuals that develop higher pT73‐Rab10 to total Rab10 ratios fare worse in disease than individuals with lower ratios according to MDS‐UPDRS and MoCA clinical scales. These results present some of the first insights regarding the population variability of peripheral phosphorylated Rabs and how they might change over the course of iPD.

Previous work identified increased autophosphorylated pS1292‐LRRK2 levels in urinary exosome fractions from G2019S *LRRK2* carriers with PD.^18^ Here, an increase in phospho‐Rab10 levels was not observed in G2019S *LRRK2* carriers with PD, despite a large body of evidence suggesting Rab10 as a genuine LRRK2 kinase substrate. Biochemical studies in transfected cells and transgenic mice suggest the G2019S *LRRK2* mutation imparts a large increase (eg, >3‐ to 4‐fold increase over WT LRRK2) in pS1292‐LRRK2 autophosphorylation levels, but has a much lower (or no) impact on phospho‐Rab levels.^5^, ^6^, ^29^ These results may be consistent with the analysis here of kidney lysates from homozygous G2019S *LRRK2* knockin mice that revealed little differences in phospho‐Rab10 levels compared with lysates from non‐transgenic controls. We have previously speculated that autophosphorylated pS1292‐LRRK2, increased by the G2019S *LRRK2* mutation, may have lower affinity with GTP‐bound Rab substrates, with LRRK2 autophosphorylation a trigger for dissociation from the membrane.^6^, ^7^ Further biochemical investigation might be required to understand the role, if any, of Rab10 phosphorylation in G2019S *LRRK2*‐linked disease.

The observed positive association of the ratio of pT73‐Rab10 to total Rab10 in our study with clinical scales commonly used to assess severity in iPD may be explained in several potential ways. First, the ratio may be reactive or coincidental to worsening disease in more severely progressing individuals. In this case, therapeutic normalization of the phosphorylation, for example through LRRK2 kinase inhibition, may not change disease course. Alternatively, the ratio measured in urine may reflect part of a specific systemic LRRK2‐dependent disease process driving worse disease in some individuals. In this case, normalization of the phosphorylation may positively impact disease progression. However, the ratio of pT73‐Rab10 to total Rab10 does not appear effective as a diagnostic biochemical marker that might separate cases from controls. This may reflect aspects of disease heterogeneity within iPD that are not well understood where LRRK2 kinase activity may play a larger role in disease onset and progression in some individuals with iPD compared to others. Further investigations in larger cohorts with biochemical, genetic, and environmental exposure integration may be revealing in this regard.

This study adopted the relatively low‐throughput method of ultra‐centrifugation of relatively large volumes of urine biospecimens, and laborious immunoblots to analyze the proteins of interest. As opposed to serum or cerebrospinal fluid (CSF), normalization of markers in urine is typically a notable challenge, because some urine donations from subjects can be incredibly dilute. In this case, in our de novo effort to measure pT73‐Rab10 in urine samples, we used the ratio of phospho to total protein from relatively large volumes of urine to eliminate the need for normalization to a different arbitrary housekeeping protein or other factor in urine (eg, creatinine) that may confound analysis. Indeed, urine represents an ideal biofluid matrix in this case that can be collected from subjects non‐invasively and at home. Although we did not have access to an established recombinant phospho‐Rab10 standard for this study from which to extract the absolute amounts of phosphorylated and total Rab10 protein in different samples, even if we applied a conversion to change the ratios here into an absolute percentage of Rab10 that was phosphorylated, we would not expect a change in results. Because we detected increased pT73‐Rab10 to total Rab10 ratios in later collection points in cases, but not in controls that were collected at the same time, we do not expect freezer storage time of the biobanked urine to have significantly skewed the results. As is typical from a single cohort analyzed, the findings here require replication in another cohort and further exploration in other biofluids like CSF, plasma, and possibly immune cell subpopulations associated with high LRRK2 expression and activity. The assay precision afforded by Western blot might be limiting in future studies, where higher throughput assays with more precision may facilitate additional insights. Single‐molecule based assays have been successfully developed for very low concentrations of phospho‐tau proteins in biofluids that may obviate the need for the purification of P100 fractions of urine as well as Western blotting.^30^ Similar assays may have use in the detection of phospho‐Rab proteins.

It is not known, which tissues and cells might contribute Rab10 protein to the vesicle‐enriched fraction of urine, because proteins in urine might come from across the body,^15^ and the biological reason for elevated pT73‐Rab10 to total Rab10 ratios in a proportion of iPD cases was not identified. The specific genome‐wide association study (GWAS) variants in *LRRK2* that are over‐represented in iPD, for example rs76904798, are rare (MAF ~0.1) and present in only a few subjects in this study. Because *LRRK2* is linked to iPD, but also to other diseases of inflammation including Crohn's disease and mycobacterial infection, it could be that late systemic inflammation in worsening disease may manifest in increased LRRK2 activity and increased phosphorylated Rab10 (eg, responsive to aggregated α‐synuclein as we detected recently in monocytes).^31^ Evaluation of LRRK2 markers in other diseases besides iPD would be informative in this regard. Some evidence for systemic LRRK2 changes driving central responses in the brain via inflammation has been observed in mouse models.^32^ As LRRK2 inhibitors move forward in clinical trials, biomarkers like Rab10 may facilitate identification of iPD groups that might benefit from LRRK2‐targeting therapies.

## Financial Disclosures Additional financial disclosures and conflicts of interest unrelated to the current research

A.B.W is a current member of the Michael J. Fox Executive Scientific Advisory Board and paid consultant for EscapeBio and Neuro23 and has received research grants unrelated to this study from Biogen, Pfizer, and EscapeBio, as well as MJFF, ASAP foundation, Parkinson Foundation, and NIH/NINDS. A.B.W. is part owner of a series of LRRK2 kinase inhibitor (WO 2013166276) and part owner of induced‐pluripotent stem cell lines distributed by Cedars Sinai. T.M.D. is a consultant to Inhibikase Therapeutics; T.M.D. serves on the Board of Directors and is compensated for his roles as a consultant to Valted Seq. T.M.D. owns stock in American Gene Technologies International.; T.M.D owns stock and stock options in Mitokinin; T.M.D. own stock options in Inhibikase Therapeutics; T.M.D. is a founder of Valted, and holds an ownership equity interest in the company. T.M.D. is an inventor of technology of Neuraly. that has optioned from Johns Hopkins University. T.M.D. is a founder of, and holds shares of stock options as well as equity in, Neuraly, which is now a subsidiary of D&D Pharmatech; T.M.D. holds shares of stock options as well as equity in D&D Pharmatech; T.M.D. is a founder of and hold equity in Valted Seq. These arrangements have been reviewed and approved by the Johns Hopkins University in accordance with its conflict of interest policies. A.P. receives funding from the NIH (NINDS/NIA), is on the Scientific Advisory Board of MedRhythms and has been compensated for his role as consultant to SciNeuro. L.S.R. received funding from the NIH (NINDS), the Michael J. Fox Foundation, National Ataxia Foundation, and the Gordon and Marilyn Macklin Foundation and has been compensated for her role on an advisory board for Bial Biotech. Other authors have nothing to report.

## Author Roles

(1) Research project: A. Conception, B. Organization, C. Execution; (2) Statistical Analysis: A. Design, B. Execution, C. Review and Critique; (3) Manuscript: A. Writing of the First Draft, B. Review and Critique.

S.W. and A.B.W designed the study and analyzed data, S.W., S.U., and H.A. performed statistical analyses, N.B. performed mouse studies, A.C. performed immunoblot measurements and exosome purification from coded samples, L.S.R., A.P., and T.M.D. established the cohorts and collected samples, and all authors drafted the manuscript.

## Supporting information


**Figure S1** Pool intensity variability and intra‐subject variability. (A) Raw intensity values for the measured JH‐PDBP pool sample for pT73‐Rab10 or (B) total Rab10 protein. Values given relate to chemiluminescent intensity values present on different membranes used to calculate groups of samples. Variability reflected in the different values are likely because of technical variability in loading the same sample in different gels, differences in gel to membrane protein transfer efficiency, antibody concentration variance and effective binding on the membrane, and ECL substrate exposure, room temperature variance, and incubation times. The observed SD for pT73‐Rab10 from different runs of the same pool was 17.5% (A) and for total Rab10 protein 12.8% (B). (C) Within‐subject variation through the course of the study for pT73‐Rab10 to total Rab10 protein. Two high dots in the iPD group (31.4 and 18.0 in the iPD group) are not shown in the plot.

**Table S1** MJFF LRRK2 Cohort participant baseline characteristics

**Table S2** Demographics and selected clinical information for the Johns Hopkins Parkinson's disease biomarker program (JH‐PDBP) cohort.

## Data Availability

Data available on request from the authors
