# Supplementary material for: Elevated Urinary Rab10 Phosphorylation in Idiopathic Parkinson Disease
Source: Mov Disord. 2022 May 6;37(7):1454–64. doi: 10.1002/mds.29043 (PMC9308673; doi:10.1002/mds.29043)
Supplement: Supplementary file 1 — Figure S1 Pool intensity variability and intra‐subject variability. (A) Raw intensity values for the measured JH‐PDBP pool sample for pT73‐Rab10 or (B) total Rab10 protein. Values given relate to chemiluminescent intensity values present on different membranes used to calculate groups of samples. Variability reflected in the different values are likely because of technical variability in loading the same sample in different gels, differences in gel to membrane protein transfer efficiency, antibody concentration variance and effective binding on the membrane, and ECL substrate exposure, room temperature variance, and incubation times. The observed SD for pT73‐Rab10 from different runs of the same pool was 17.5% (A) and for total Rab10 protein 12.8% (B). (C) Within‐subject variation through the course of the study for pT73‐Rab10 to total Rab10 protein. Two high dots in the iPD group (31.4 and 18.0 in the iPD group) are not shown in the plot. [file MDS-37-1454-s002.docx]

**
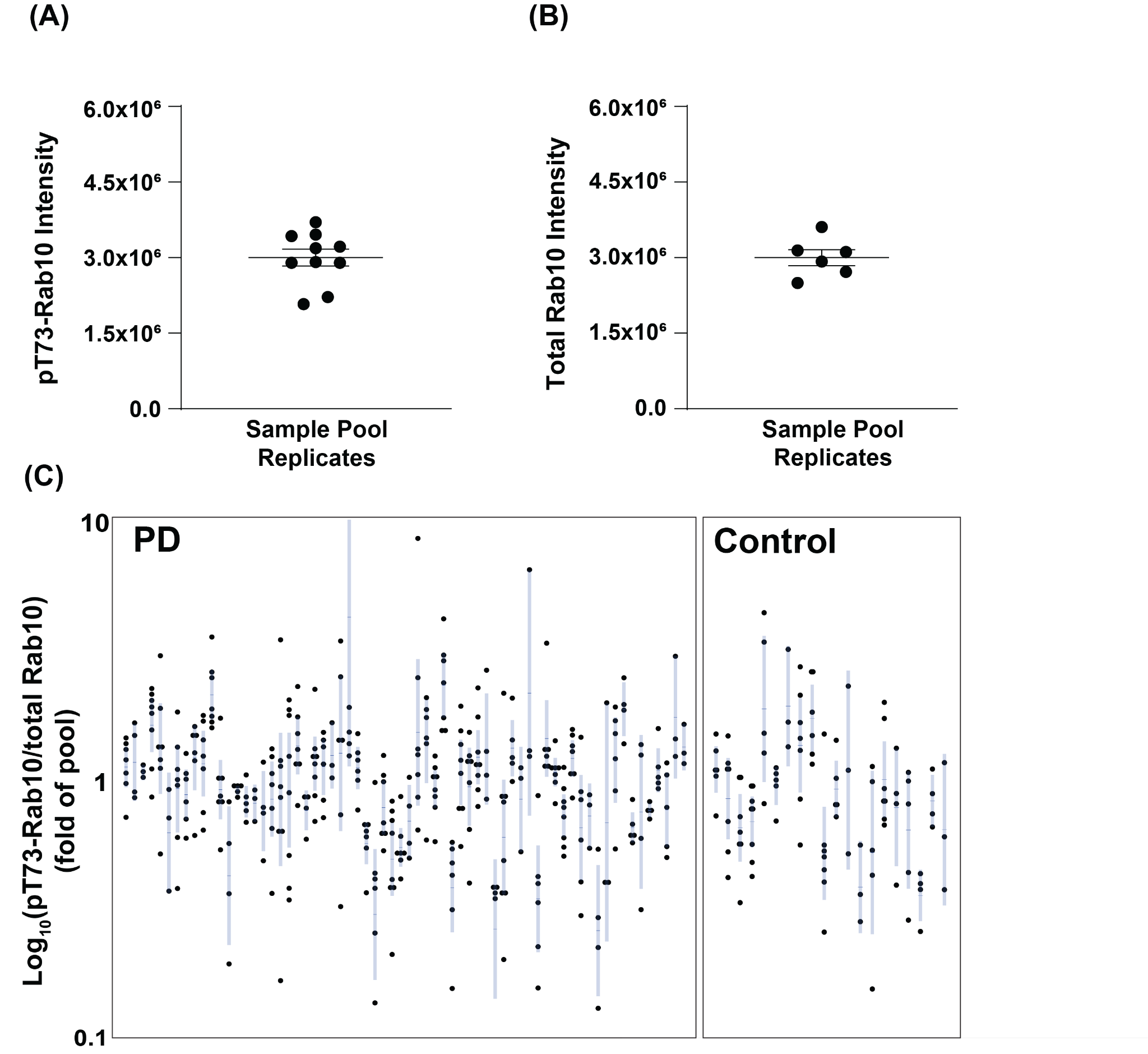
**

**Supplemental Figure 1.** Pool intensity variability and intra-subject variability. A. Raw intensity values for the measured JH-PDBP pool sample for pT73-Rab10 or B. total Rab10 protein. Values given relate to chemiluminescent intensity values present on different membranes used to calculate groups of samples. Variability reflected in the different values are likely due to technical variability in loading the same sample in different gels, differences in gel to membrane protein transfer efficiency, antibody concentration variance and effective binding on the membrane, and ECL substrate exposure, room temperature variance, and incubation times. The observed SD for pT73-Rab10 from different runs of the same pool was 17.5% (panel A) and for total Rab10 protein 12.8% (panel B). C. Within-subject variation through the course of the study for pT73-Rab10 to total Rab10 protein. Two high dots in the iPD group (31.4 and 18.0 in the iPD group) are not shown in the plot.
