# Supplementary material for: Elevated Urinary Rab10 Phosphorylation in Idiopathic Parkinson Disease
Source: Mov Disord. 2022 May 6;37(7):1454–64. doi: 10.1002/mds.29043 (PMC9308673; doi:10.1002/mds.29043)
Supplement: Supplementary file 2 — Table S1 MJFF LRRK2 Cohort participant baseline characteristics [file MDS-37-1454-s003.docx]

**Supplemental Table 1.** MJFF LRRK2 Cohort participant baseline characteristics

| **Characteristic** | **LRRK2-PD-**  **(N=12)** | **LRRK2-PD+ (N=17)** | **LRRK2+PD+ (N=16)** |
| --- | --- | --- | --- |
| Age (Yrs.) |  |  |  |
| n | 11* | 17 | 16 |
| Mean (SD) | 62.6 (15.2) | 65.4 (8.5) | 68.9 (7.9) |
| Median (q1,q3) | 63.0 (49, 77) | 62 (60.5, 71.5) | 69 (62, 75) |
| Min/Max | 42 / 89 | 50 / 81 | 55 / 82 |
| Age at Diagnosis (Yrs.) |  |  |  |
| n | NA | 17 | 16 |
| Mean (SD) | NA | 55.8 (8.7) | 57.1 (11.5) |
| Median (q1,q3) | NA | 56.0 (49.5, 63.0) | 59.5 (45.75, 67.0) |
| Min/Max | NA | 39.0 / 69.0 | 36.0 / 75.0 |
| Disease Duration (Yrs.) |  |  |  |
| n | NA | 17 | 16 |
| Mean (SD) | NA | 9.5 (5.3) | 11.0 (5.0) |
| Median (q1,q3) | NA | 8.0 (5.0, 13.0) | 9.0 (7.0, 15.75) |
| Min/Max | NA | 2.0 / 20.0 | 4.0 / 19.0 |
| L-dopa Equivalent Daily Dosage (LEDD) |  |  |  |
| n | NA | 17 | 16 |
| Mean (SD) | NA | 637.9 (370.4) | 643.4 (568.3) |
| Median (q1,q3) | NA | 700.0 (325.0, 950.0) | 525.0 (262.5, 850.0) |
| Min/Max | NA | 0.0 / 1340.0 | 0.0 / 2280.0 |
| MoCA Total |  |  |  |
| n | 12 | 17 | 16 |
| Mean (SD) | 25.8 (2.9) | 26.8 (2.9) | 26.1 (3.7) |
| Median (q1,q3) | 26.5 (23.5, 28.0) | 28.0 (25.5, 29.0) | 26.0 (25.0, 28.75) |
| Min/Max | 20.0 / 29.0 | 21.0 / 30.0 | 14.0 / 30.0 |
| ESS Score: |  |  |  |
| n | 12 | 17 | 16 |
| Mean (SD) | 3.9 (3.7) | 9.1 (5.5) | 8.3 (2.7) |
| Median (q1,q3) | 2.5 (1.0, 6.0) | 9.0 (3.5, 15.0) | 8.0 (6.25, 9.75) |
| Min/Max | 0.0 / 11.0 | 2.0 / 18.0 | 5.0 / 15.0 |
| H&Y Score: |  |  |  |
| n | 12 | 17 | 16 |
| Mean (SD) | 0.0 (0.0) | 2.3 (0.5) | 1.9 (0.7) |
| Median (q1,q3) | 0.0 (0.0, 0.0) | 2.0 (2.0, 2.5) | 2.0 (2.0, 2.0) |
| Min/Max | 0.0 / 0.0 | 0.0 / 4.0 | 0.0 / 3.0 |
| UPSIT Score: |  |  |  |
| n | 12 | 17 | 16 |
| Mean (SD) | 28.7 (11.4) | 14.6 (8.8) | 18.75 (13.0) |
| Median (q1,q3) | 33.5 (20.75, 36.75) | 34.5 (28.0, 38.0) | 19.5.0 (6.25, 29.5) |
| Min/Max | 8.0 / 38.0 | 8.0 / 38.0 | 0.0 / 39.0 |
| Total LRRK2 (fold of pool) |  |  |  |
| n | 12 | 17 | 16 |
| Mean (SD) | 0.38 (0.37) | 1.24 (1.51) | 1.52 (1.60) |
| Median (q1,q3) | 0.31 (0.19, 0.37) | 0.59 (0.20, 2.30) | 0.77 (0.58, 2.08) |
| Min/Max | 0.02 / 1.46 | 0.03 / 5.77 | 0.22 / 6.06 |
| pT73-Rab10 / Total Rab10 (fold of pool) | |  |  |
| n | 12 | 17 | 16 |
| Mean (SD) | 0.24 (0.14) | 0.22 (0.11) | 0.26 (0.13) |
| Median (q1,q3) | 0.21 (0.11, 0.30) | 0.19 (0.13, 0.24) | 0.23 (0.16, 0.32) |
| Min/Max | 0.10 / 0.50 | 0.09 / 0.43 | 0.13 / 0.54 |

LRRK2+: G2019S LRRK2; ESS: Epworth SS, Epworth Sleepiness Scale; MoCA, The Montreal Cognitive Assessment; H&Y: Hoehn and Yahr Stage (modified); LEDD, L-dopa equivalent; UPSIT, University of Pennsylvania Smell Identification Test.

*Age was not recorded in the database for the subjects older than 89.
