# Supplementary material for: Elevated Urinary Rab10 Phosphorylation in Idiopathic Parkinson Disease
Source: Mov Disord. 2022 May 6;37(7):1454–64. doi: 10.1002/mds.29043 (PMC9308673; doi:10.1002/mds.29043)
Supplement: Supplementary file 3 — Table S2 Demographics and selected clinical information for the Johns Hopkins Parkinson's disease biomarker program (JH‐PDBP) cohort. [file MDS-37-1454-s001.docx]

**Supplemental Table 2.** Demographics and selected clinical information for the Johns Hopkins Parkinson’s disease biomarker program (JH-PDBP) cohort.

| **Characteristic** | **Case, N = 90** | **Control, N = 37** | **Combined, N = 127** |
| --- | --- | --- | --- |
| **Age (Yrs.)** |  |  |  |
| Mean (SD) | 65.9 (8.1) | 67.0 (8.7) | 66.2 (8.3) |
| Median (q1,q3) | 67.0 (60.0, 70.8) | 67.6 (64.6, 71.3) | 67.0 (60.9, 70.9) |
| Min/Max | 41.5 / 87.5 | 46.1 / 83.0 | 41.5 / 87.5 |
| **Study Visits** |  |  |  |
| Mean (SD) | 4.9 (2.8) | 4.8 (2.8) | 4.8 (2.8) |
| **Observation length (Months)** |  |  |  |
| Mean (SD) | 19.0 (15.9) | 20.5 (15.3) | 20.2 (15.4) |
| **Age at Diagnosis (Yrs.)** |  |  |  |
| Mean (SD) | 59.3 (8.6) | NA | 59.3 (8.6) |
| Median (q1,q3) | 59.5 (53.0, 66.0) | NA | 59.5 (53.0, 66.0) |
| Min/Max | 40.0 / 82.0 | NA | 40.0 / 82.0 |
| **Disease Duration (Yrs.)** |  |  |  |
| Mean (SD) | 6.6 (4.8) | NA | 6.6 (4.8) |
| Median (q1,q3) | 5.4 (3.0, 8.9) | NA | 5.4 (3.0, 8.9) |
| Min/Max | 0.7 / 25.5 | NA | 0.7 / 25.5 |
| **Gender** |  |  |  |
| Female | 29 / 90 (32.2%) | 26 / 37 (70.3%) | 55 / 127 (43.3%) |
| Male | 61 / 90 (67.8%) | 11 / 37 (29.7%) | 72 / 127 (56.7%) |
| **LEDD** |  |  |  |
| Mean (SD) | 728.2 (491.3) | NA | 728.2 (491.3) |
| Median (q1,q3) | 600.0 (400.0, 925.0) | NA | 600.0 (400.0, 925.0) |
| Min/Max | 50.0 / 2324.0 | NA | 50.0 / 2324.0 |
| **MDS-UPDRS Part I Score:** |  |  |  |
| Mean (SD) | 11.9 (6.4) | 5.6 (5.6) | 10.0 (6.8) |
| Median (q1,q3) | 10.5 (7.0, 17.0) | 3.0 (2.0, 8.0) | 9.0 (5.0, 14.0) |
| Min/Max | 2.0 / 37.0 | 0.0 / 25.0 | 0.0 / 37.0 |
| **MDS-UPDRS Part II Score:** |  |  |  |
| Mean (SD) | 12.3 (8.2) | 0.9 (2.4) | 9.0 (8.7) |
| Median (q1,q3) | 10.0 (7.0, 16.0) | 0.0 (0.0, 1.0) | 7.0 (2.0, 13.0) |
| Min/Max | 1.0 / 43.0 | 0.0 / 13.0 | 0.0 / 43.0 |
| **MDS-UPDRS Total Score** |  |  |  |
| Mean (SD) | 59.7 (24.1) | 8.5 (10.6) | 44.8 (31.5) |
| Median (q1,q3) | 56.0 (46.0, 66.0) | 5.0 (3.0, 10.0) | 48.0 (12.0, 62.0) |
| Min/Max | 20.0 / 137.0 | 0.0 / 59.0 | 0.0 / 137.0 |
| **MoCA Total** |  |  |  |
| Mean (SD) | 25.5 (4.5) | 27.8 (1.2) | 26.2 (4.0) |
| Median (q1,q3) | 26.0 (24.0, 28.0) | 28.0 (27.0, 29.0) | 27.0 (25.0, 28.0) |
| Min/Max | 6.0 / 30.0 | 25.0 / 30.0 | 6.0 / 30.0 |
| MoCA, The Montreal Cognitive Assessment; MDS-UPDRS, Movement Disorders Society-Unified Parkinson's Disease Rating Scale. LEDD, L-dopa Equivalent Daily Dosage. | | | |
